# Clinicopathological Features of Programmed Cell Death-ligand 1 Expression in Patients with Oral Squamous Cell Carcinoma

**DOI:** 10.1515/med-2020-0041

**Published:** 2020-04-17

**Authors:** Yong-Xin Cui, Xian-Shuang Su

**Affiliations:** 1 Department of Stomatology, The Second Hospital of Shandong University, Jinan, 250033, China

**Keywords:** Oral squamous cell carcinom, Programmed cell death-ligand 1, Clinicopathological features, Meta-analysis

## Abstract

**Objective:**

Programmed cell death-ligand 1 (PD-L1) expression has been shown to play important roles in various types of cancer. However, the role of PD-L1 expression has not been conclusively reported in patients with oral squamous cell carcinoma (OSCC). Accordingly, in this meta-analysis, we investigated the clinicopathological value of PD-L1 expression in patients with OSCC.

**Methods:**

Google Scholar, PubMed, EMBASE, and CNKI databases were searched to find relevant studies published through to September 16, 2019. The relationships between PD-L1 expression in patients with OSCC and clinicopathological features were assessed using risk ratio (RR) and 95% confidence intervals (CIs).

**Results:**

Sixteen studies including 1989 participants were included. The results indicated that high PD-L1 expression was correlated with sex (RR = 1.28, 95% CI: 1.16–1.42, P < 0.001), N stage (RR = 1.19, 95% CI: 1.06–1.33, P = 0.003), M stage (RR = 1.64, 95% CI: 1.01–2.66, P = 0.044), low differentiation (RR = 1.16, 95% CI: 1.01–1.33, P = 0.034), and human papilloma virus infection (RR = 1.38, 95% CI: 1.14–1.68, P = 0.001), but unrelated to TNM stage or T stage. There was no significant publication bias in the studies included in this analysis.

**Conclusions:**

This meta-analysis revealed that high PD-L1 expression in patients with OSCC was correlated with clinicopathological features. Further large-scale studies are necessary to confirm our results.

## 1 Introduction

Oral cancer is a major public health concern worldwide; approximately 350,000 patients are newly diagnosed with oral cancer each year, and oral cancer causes approximately 170,000 deaths annually [1]. Oral squamous cell carcinoma (OSCC) accounts for nearly 90% of malignant oral carcinomas, and the 5-year survival rate is only approximately 50% [2, 3]. Owing to the high rate of metastasis in patients with OSCC, the prognosis tends to be poor [4]. Prediction of prognosis plays a critical role in the treatment of OSCC and is usually based on the tumor-node-metastasis (TNM) classification system; lymph node metastases and the presence of distant metastases are associated with a poor prognosis [5, 6]. Despite recent advancements in various therapies, including radiotherapy, chemotherapy, and surgery, the survival rates of patients with OSCC have not improved [2]. Thus, the identification of novel prognostic markers is urgently needed to improve personalized treatment approaches and clinical outcomes in patients with OSCC.

Programmed cell death-ligand 1 (PD-L1), also known as B7-H1 or CD274, is a member of the costimulatory factor superfamily [7]. PD-L1 is expressed in various types of tumor cells and in immune cells, including activated B cells and T cells, macrophages, and dendritic cells [8]. When the programmed cell death-1 (PD-1)/PD-L1 axis is highly expressed in a healthy immune system, activation of this pathway restricts autoimmunity and limits T-cell activity in an inflammatory response to infection [9]. In contrast, overexpression of PD-L1 in carcinoma cells blocks the activation of T cells, exhausts T cells, and triggers apoptosis in effector T cells, thereby impairing cytokine production and promoting tumor growth [10, 11, 12].

Previous studies have reported the prognostic value of PD-L1 expression in many types of malignant solid tumors, such as pancreatic carcinoma [13], non-small cell lung carcinoma [14], prostate cancer [15], gastric carcinoma [16], and breast cancer [17]. Moreover, although several studies have investigated the associations between PD-L1 expression and clinicopathological characteristics in patients with OSCC, the results remain contradictory [18, 19, 20, 21, 22, 23, 24, 25, 26, 27, 28, 29, 30, 31, 32]. For example, Straub and colleagues found that PD-L1 overexpression is closely related to lymph node metastasis and is correlated with poor overall survival in patients with OSCC [24]. In contrast, Hong et al. revealed that high PD-L1 expression is associated with better prognosis in patients with OSCC [19]. In a study by Cho and colleagues, however, PD-L1 expression does not affect survival rates in patients with OSCC [32].

In this study, in order to clarify the role of PD-L1 in OSCC, we performed a meta-analysis of PD-L1 expression and clinicopathological features in patients with OSCC.

## 2 Methods

### 2.1 Literature search

A systematic literature search was performed of PubMed, EMBASE, Google Scholar, and CNKI up to September 16, 2019 using the following search terms: (“mouth” OR “oral”) AND (“carcinoma” OR “tumor” OR “neoplasm” OR “cancer”) AND (“B7-H1” OR “programmed cell death ligand 1” OR “PD-L1”). The study was performed according to the Statement of the Preferred Reporting Items for Systematic Reviews and Meta-Analyses [33].

### 2.2 Inclusion and exclusion criteria

The included studies met the following inclusion criterion: (a) participants were histologically diagnosed with OSCC; (b) articles were written in English or Chinese with full text available, and humans were used as the study subjects; (c) the expression level of the *PD-L1* gene was estimated in OSCC tissues; (d) the relationship of PD-L1 expression with clinicopathological features was investigated in OSCC patients; (e) studies had sufficient materials to estimate relative risk (RR) with corresponding 95% confidence intervals (95% CIs). Exclusion criteria were as follows: (a) reviews, editorials, conference abstracts, and case reports; and (b) studies that had insufficient data.

### 2.3 Data extraction and quality assessment

The available data for the included studies were independently extracted by two authors. The following data were extracted: first author, country, ethnicity, publication year, detection method, and clinicopathological parameters. Disagreement was settled through discussion between authors. The Newcastle-Ottawa-Scale (NOS) was applied to estimate the quality of the included studies [34].

### 2.4 Statistical analysis

The relationships between PD-L1 expression in patients with OSCC and clinicopathological characteristics were assessed using RR and 95% CIs. Cochrane’s *Q* tests and the I^2^ statistic were carried out to evaluate between-study heterogeneity. Significant heterogeneity was defined as *P* < 0.1 or I^2^ > 50%, and RR were then pooled using the random-effect model [35]; Or else, a fixed-effect model was chosen [36]. Additionally, we performed a sensitivity analysis to determine the stability of the pooled values. To estimate potential publication bias, Egger linear regression tests and Begg’s funnel plots were used [37, 38]. All analyses were performed using Stata 15.0 software (Stata Corp., College Station, TX, USA).

## 3 Results

### 3.1 Literature search results

**Figure 1:**
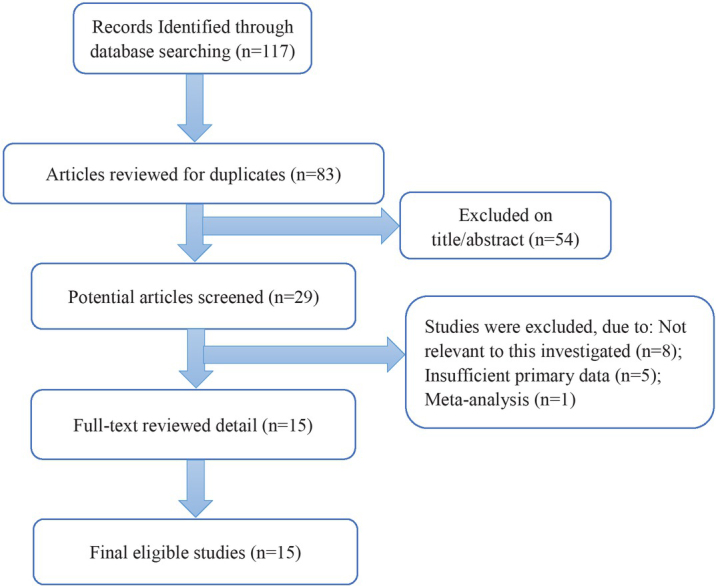
Flow chart of study identification.

Figure 1 shows the literature search process. In total, 117 studies were selected from our database search. Duplicates were deleted, 83 articles were screened, and 54 records were further removed. The full text of the remaining 29 articles was read. Finally, 15 articles were included in the current analysis [18, 19, 20, 21, 22, 23, 24, 25, 26, 27, 28, 29, 30, 31, 32].

### 3.2 Description of the included studies

Sixteen retrospective studies including 1989 participants were included in our meta-analysis of the association between PD-L1 expression and clinicopathological features in patients with OSCC. Among the 15 articles, data describing sex (1947 patients; female versus male), T stage (1768 patients; T3/T4 versus T1/T2), N stage (1663 patients; N1–N3 versus N0), M stage (581 patients; M1 versus M0), TNM stage (1351 patients; III/IV versus I/II), histological grade (1486 patients; poorly/moderately versus well differentiated), recurrence status (333 patients; yes versus no), and human papilloma virus (HPV) status (935 patients; positive versus negative) were included. Among the 16 studies, eight studies evaluated Asians, and eight studies evaluated Caucasians. The total sample size was 1989, ranging from 24 to 305. The included articles were published between 2011 and 2019. The expression level of PD-L1 in patients with OSCC was detected using immuno-histochemistry. The quality of the included studies was evaluated by the NOS, and the scores for the included literature ranged from 6 to 9, indicating that the enrolled studies were of a relatively high quality. Detailed information for the included studies is presented in Table 1.

**Table 1:** Characteristics of included studies.

					TNM		PD-L1 expression		
Author	Year	Country	Ethnicity	Total (n)	stage	Detection method	Positive	Negative	NOS
Cho	2011	Korea	Asian	45	I-IV	IHC	26	19	6
Ukpo	2013	USA	Caucasian	181	I-IV	IHC	84	97	7
Lin	2015	China	Asian	305	I-IV	IHC	133	172	7
Oliveira-Costa	2015	Brazil	Caucasian	142	I-III	IHC	47	49	8
Hong	2016	Australia	Caucasian	99	I-IV	IHC	69	30	7
Kim	2015	Korea	Asian	133	I-IV	IHC	90	43	9
Ock(a)	2016	Korea	Asian	50	I-IV	IHC	32	18	8
Ock(b)	2016	Korea	Asian	91	I-IV	IHC	59	32	8
Satgunaseelan	2016	Australia	Caucasian	217	NA	IHC	40	177	9
Straub	2016	Germany	Caucasian	80	I-IV	IHC	36	44	7
Meulenaere	2017	Belgium	Caucasian	99	I-IV	IHC	22	72	8
Hirai	2017	Japan	Asian	24	I-IV	IHC	13	11	7
Kogashiwa	2017	Japan	Asian	84	I-IV	IHC	44	40	7
Troeltzsch	2016	Germany	Caucasian	88	I-IV	IHC	26	62	8
Hong	2019	Australia	Caucasian	214	I-IV	IHC	145	69	9
Sato	2019	Japan	Asian	137	I-IV	IHC	81	56	8

PD-L1, programmed cell death ligand 1; NA, not available; IHC, immunohistochemical; NOS, Newcastle-Ottawa-Scale.

### 3.3 Meta-analysis results

#### 3.3.1 Sex

**Figure 2:**
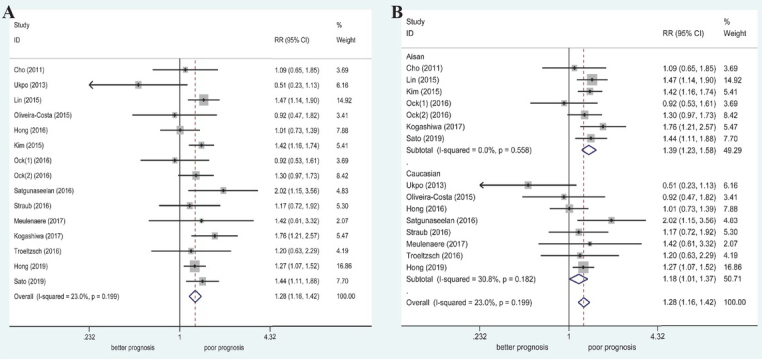
Forest plot of RRs and 95% CIs for the association between PD-L1 expression and sex. (A) Overall population; (B) stratified by ethnicity.

Fifteen studies (1947 patients; 458 women and 1489 men) were included for evaluation of the relationship between PD-L1 expression and sex in patients with OSCC. There was a low degree of heterogeneity among the studies (I^2^ = 23.0%, *P* = 0.199); thus, the fixed-effect model was used for pooled analysis. The results indicated a statistically significant relationship between high PD-L1 expression and female sex (RR = 1.28, 95% CI: 1.16–1.42, *P* < 0.001). Subgroup analysis by race indicated that high PD-L1 expression was associated significantly with women in both Caucasian and Asian populations (Table 2 and Figure 2).

**Table 2:** Correlation between clinical variables and PD-L1 expression in OSCC.

		Heterogeneity		Association			
Clinical variables	Studies	I2	p-value	RR	95%CI	p-value	Egger’s
**Gender**	14	23	0.199	1.28	1.16-1.42	<0.001	0.203
Asian	6	0	0.558	1.39	1.23-1.58	<0.001	
Caucasian	8	30.8	0.182	1.18	1.01-1.37	0.039	
**T stage**	14	13.2	0.309	1.03	0.94-1.13	0.546	0.879
Asian	6	0	0.9	0.93	0.80-1.07	0.295	
Caucasian	8	29.1	0.196	1.12	0.98-1.27	0.094	
**N stage**	13	40.6	0.063	1.19	1.06-1.33	0.003	0.575
Asian	5	0	0.639	0.98	0.82-1.18	0.845	
Caucasian	8	40.4	0.109	1.34	1.16-1.56	<0.001	
**M stage**	3	68.7	0.041	1.64	1.01-2.66	0.044	0.12
**TNM stage**	9	49.6	0.037	0.9	0.75-1.08	0.252	0.264
Asian	5	63.5	0.018	0.92	0.70-1.20	0.537	
Caucasian	4	19.2	0.294	0.86	0.68-1.10	0.235	
**Differentiation**	11	0	0.624	1.16	1.01-1.33	0.034	0.942
Asian	4	0	0.72	1.16	0.94-1.43	0.168	
Caucasian	7	13	0.331	1.16	0.97-1.39	0.106	
**Recurrence**	4	0	0.585	0.75	0.57-1.00	0.046	0.291
**HPV-Positive**	7	59.6	0.015	1.38	1.14-1.68	0.001	0.953
Asian	2	0	0.713	1.22	1.02-1.46	0.011	
Caucasian	5	70.8	0.008	1.54	1.10-2.14	0.027	

PD-L1, programmed cell death ligand 1; CI, confidence interval; RR, relative risks.

#### 3.3.2 N stage

**Figure 3:**
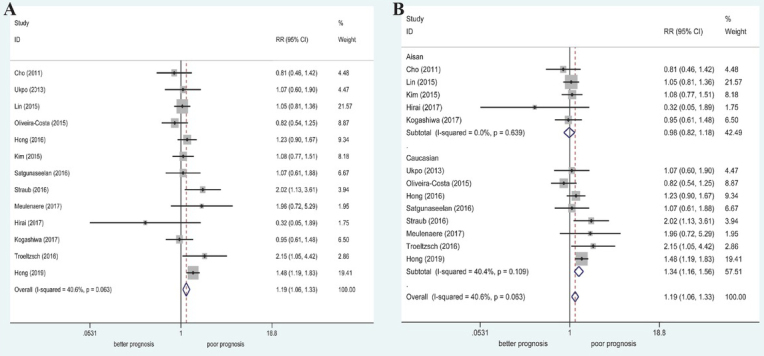
Forest plot of RRs and 95% CIs for the association between PD-L1 expression and N stage. (A) Overall population; (B) stratified by ethnicity.

Thirteen studies (1663 patients; 958 with N1–N3 stage and 705 with N0 stage) were included for evaluation of the relationship between PD-L1 expression and lymph node metastasis in patients with OSCC. Moderate heterogeneity was found among the studies (I^2^ = 40.6%, P = 0.063); thus, the fixed-effect model was used for pooled analysis. The results indicated that there was a significant relationship between high PD-L1 expression and lymph node metastasis (N1–N3; RR = 1.19, 95% CI: 1.06–1.33, P = 0.003). Subgroup analysis by race indicated that high PD-L1 expression was significantly correlated with lymph node metastasis among Caucasians (RR = 1.34, 95% CI: 1.16–1.56, P < 0.001; Table 2 and Figure 3).

#### 3.3.3 Histological grade

**Figure 4:**
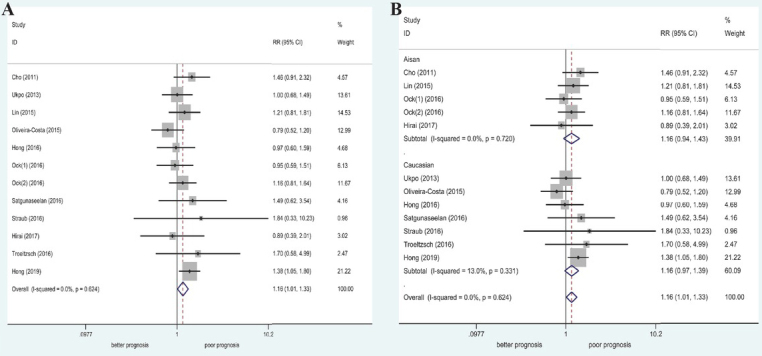
Forest plot of RRs and 95% CIs for the association between PD-L1 expression and histological grade. (A) Overall population; (B) stratified by ethnicity.

Twelve studies (1486 patients; 1149 with poorly/moderately differentiated disease and 337 with well differentiated disease) were included for assessment of the association between histological grade and PD-L1 expression. No significant heterogeneity was found (I^2^ = 0%, *P* = 0.624); thus, the fixed-effect model was used for pooled analysis. The results revealed a significant relationship between high PD-L1 expression and advanced histological grade (poorly/moderately differentiated; RR = 1.16, 95% CI: 1.01– 1.33, *P* = 0.034). In the stratification according to ethnicity, we found no significant relationship between high PD-L1 expression and different histological grades (Table 2 and Figure 4).

#### 3.3.4 HPV status

**Figure 5:**
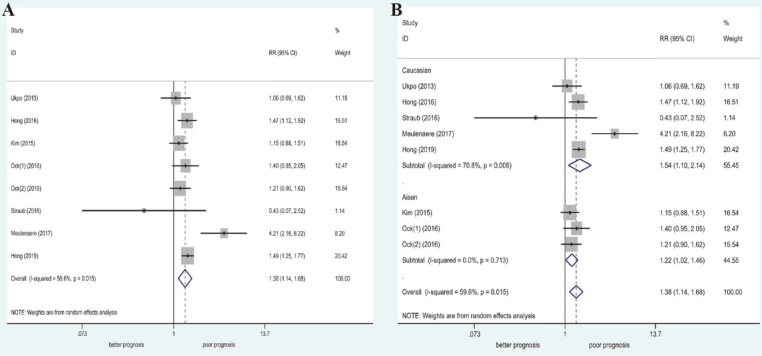
Forest plot of RRs and 95% CIs for the association between PD-L1 expression and HPV status. (A) Overall population; (B) stratified by ethnicity.

Eight studies (935 patients; 424 with HPV-associated disease and 511 without HPV-associated disease) were included for evaluation of the relationship between HPV status and PD-L1 expression. Moderate heterogeneity was found among the studies (I^2^ = 59.6%, *P* = 0.015); thus, a random-effect model was used for pooled analysis. The results demonstrated a significant association between high PD-L1 expression and HPV-associated OSCC (RR = 1.38, 95% CI: 1.14–1.68, P = 0.001). In the subgroup analysis stratified based on ethnicity, we found that high PD-L1 expression was significant correlated with HPV-as-sociated OSCC among Caucasian and Asian populations (Table 2 and Figure 5)

#### 3.3.5 Other clinicopathological features

Four studies (333 patients; 85 with recurrence and 248 without recurrence) were included for evaluation of the relationship between PD-L1 expression and recurrence status in patients with OSCC. No heterogeneity was found (I^2^ = 0%, *P* = 0.585); thus, a fixed-effect model was used for the pooled analysis. The results revealed a significant relationship between high PD-L1 expression and recurrence (RR = 0.75, 95% CI: 0.57–1.00, P = 0.046). However, high expression of PD-L1 was not significantly correlated with T stage (RR = 1.03, 95% CI: 0.94–1.13, P = 0.546) or TNM stage (RR = 0.90, 95% CI: 0.75–1.08, P = 0.252; Table 2).

### 3.4 Sensitivity analysis and publication bias

**Figure 6:**
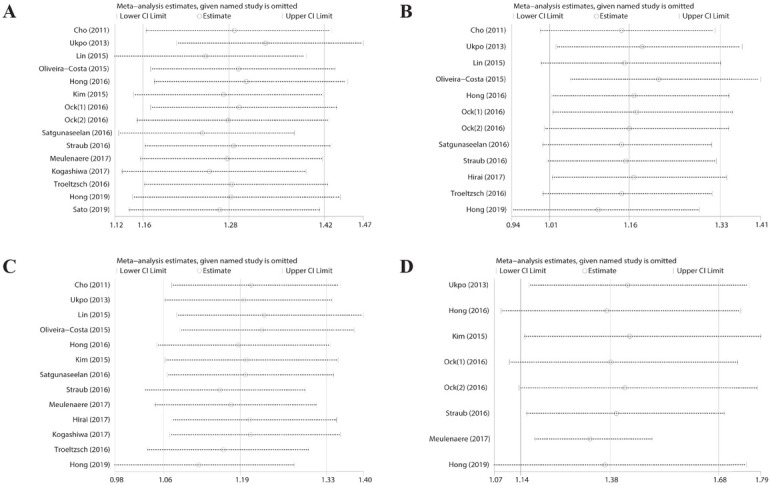
Sensitivity analysis for the association between PD-L1 expression and (A) sex; (B) grade; (C) N stage; and (D) HPV status.

**Figure 7:**
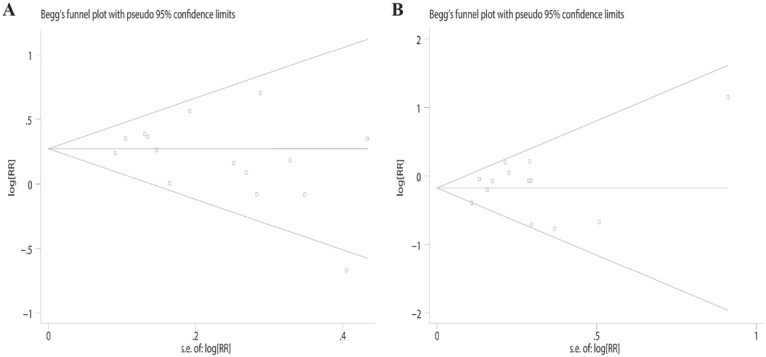
Begg’s funnel plot for the association between PD-L1 expression and (A) sex and (B) N stage.

We performed a sensitivity analysis by sequentially deleting each study individually; the results indicated that the pooled RR was unaffected, as shown in Figure 6. Potential publication bias was evaluated using Egger’s test and Begg’s funnel. Funnel plots were largely symmetric, indicating no obvious publication bias, as shown in Figure 7 and Table 2.

## 4 Discussion

PD-L1, an immunoinhibitory receptor that was first described in 1992 by Ishida, is expressed in tumor cells and various types of immune cells, including activated B cells and T cells, macrophages, and dendritic cells [8, 39]. PD-L1 is an essential regulatory molecule in the immune system and is critical for the immune escape mechanisms of many types of cancer cells [40]. Overexpression of PD-L1 results in an immunosuppressive tumor microenvironment and prevents T cells from mediating cytolysis in numerous solid tumors. In some tumor cells, PD-L1 blocks the activation of T cells, exhausts T cells, triggers apoptosis in effector T cells, and impairs cytokine production, resulting in tumor growth [10, 11, 12].

PD-L1’s immune checkpoint response has been extensively studied and plays predominant roles in immune surveillance during tumor development and immune escape of cancer cells [41]. Immune checkpoint inhibitors, including nivolumab and pembrolizumab, have been approved to treat OSCC [42, 43, 44, 45, 46]. Despite the importance of the immune checkpoint, the clinicopathological effects of PD-L1 expression in patients with OSCC remain unclear. In this study, we performed a comprehensive and systematic analysis of the clinicopathological significant of PD-L1 expression in patients with OSCC. Our findings showed that high PD-L1 expression was significantly correlated with certain clinicopathological parameters, including female sex, lymph node metastasis (N1–N3), and advanced histological grade (poorly/moderately differentiated), in patients with OSCC.

In a previous study by Lin et al., high PD-L1 expression was found to be associated with low overall survival in patients with OSCC, and PD-L1 was highly expressed in women [30], consistent with our results. However, there was no significant correlation between high PD-L1 expression and sex in patients with OSCC in another study [47]. Thus, it remains unclear whether sex plays a role in influencing PD-L1 expression in patients with OSCC. In our study, the results demonstrated that high PD-L1 expression was significantly related to lymph node metastasis and advanced histological grade, consistent with some previous studies [19, 32]. These characteristics suggest that deviations in the PD-L1 pathway in malignant tumors are associated with more malignant clinical conditions, including tumor prognosis and progression. Moreover, we also investigated the association between HPV status and high PD-L1 expression in patients with OSCC; the results showed that high PD-L1 expression was significantly related to HPV-associated OSCC, consistent with previous studies [19]. However, no significant relationship was found between the high PD-L1 expression and HPV status in a different study [31], potentially because of the limited sample size. Overall, our meta-analysis revealed that high PD-L1 expression was associated with several clinicopathological features in patients with OSCC, suggesting that PD-L1 may play a role in the clinical diagnosis and prognosis of OSCC.

There were several limitations to our current results. First, although 16 studies were selected, the sample size was relatively small, with only 1989 patients included in the evaluated studies. Second, the studies were published in Chinese and English, which may have resulted in publication bias; however, we detected no publication bias in this study. Third, significant heterogeneity was observed between studies; thus, we implemented this meta-analysis using random-effect models and sensitivity analysis to verify the reliability of our results.

## 5 Conclusions

Our current meta-analysis indicated that high PD-L1 expression in patients with OSCC was correlated with clinicopathological features, suggesting the potential roles of PD-L1 in the diagnosis and prognosis of patients with OSCC. To verify our results, further large-scale studies are needed.
